# Cytosine Deaminase-Overexpressing hTERT-Immortalized Human Adipose Stem Cells Enhance the Inhibitory Effects of Fluorocytosine on Tumor Growth in Castration Resistant Prostate Cancer

**DOI:** 10.3390/ijms25105519

**Published:** 2024-05-18

**Authors:** Jae Heon Kim, Hee Jo Yang, Sang Hun Lee, Yun Seob Song

**Affiliations:** 1 Department of Urology, Soonchunhyang University School of Medicine, Seoul 04401, Republic of Korea; 2 Department of Urology, Soonchunhyang University School of Medicine, Cheonan 31151, Republic of Korea; 3 Program in Biomedical Sciences & Engineering, Department of Biomedical Sciences, College of Medicine, Inha University, Incheon 22212, Republic of Korea

**Keywords:** stem cells, prostate cancer, cytosine deaminase

## Abstract

A promising de novo approach for the treatment of Castration-resistant prostate cancer (CRPC) exploits cell-mediated enzyme prodrug therapy comprising cytosine deaminase (CD) and fluorouracil (5-FC). The aim of this study was to determine the potential of bacterial CD-overexpressing hTERT-immortalized human adipose stem cells (hTERT-ADSC.CD) to suppress CRPC. A lentiviral vector encoding a bacterial *CD* gene was used to transfect and to generate the hTERT-ADSC.CD line. The ability of the cells to migrate selectively towards malignant cells was investigated in vitro. PC3 and hTERT-ADSC.CD cells were co-cultured. hTERT-ADSC.CD and 1 × 10^6^ PC3 cells were administered to nude mice via intracardiac and subcutaneous injections, respectively, and 5-FC was given for 14 days. hTERT-ADSC.CD were successfully engineered. Enhanced in vitro hTERT-ADSC.CD cytotoxicity and suicide effect were evident following administration of 5 μM 5-FC. hTERT-ADSC.CD, together with 5-FC, augmented the numbers of PC3 cells undergoing apoptosis. In comparison to controls administered hTERT-ADSC.CD monotherapy, hTERT-ADSC.CD in combination with 5-FC demonstrated a greater suppressive effect on tumor. In CPRC-bearing mice, tumor suppression was enhanced by the combination of CD-overexpressing ADSC and the prodrug 5-FC. Stem cells exhibiting *CD* gene expression are a potential novel approach to treatment for CRPC.

## 1. Introduction

Prostate carcinoma (PC) is the most frequently arising tumor in men, and the second leading cause of mortality resulting from malignancy. Therapy for castration-resistant prostate carcinoma (CRPC) forms the principal constraint in terms of managing this disease [1,2]. Although several novel pharmaceutical agents have entered the clinical domain, only a small number have demonstrated clinical benefits over the long-term.

Gene therapy, a de novo therapeutic approach, targeted at the cellular level, has the potential to surmount these issues, particularly in terms of reducing treatment toxicity [3,4]. A number of pre-clinical studies have reported encouraging data following the use of cell-mediated enzyme prodrug therapy (EPT), i.e., enzyme and prodrug treatment combined, against solid tumors [3,4]. One such EPT includes cytosine deaminase (CD), which diminishes the significant systemic toxic effects caused by 5-fluorouracil (5-FU) by catalyzing its conversion into 5-fluorocytosine (5-FC). The latter compound has a lower toxic profile [3,4]. In order for gene therapy to be successful, efficacious delivery of the treatment gene to the apposite target cells is imperative. Since a characteristic of stem cells is their capacity to migrate toward malignancy [5,6,7,8,9,10], inserting genes into these cell types has drawn considerable interest as a potential approach to cell-based gene delivery.

One study used 5-FC to treat murine PC cells which encoded the CD-uracil phosphoribosyl transferase fusion gene, and observed marked PC cell growth suppression [11]. Stem cell migration is selective, occurring in the direction of certain forms of malignancy [5,6,7,8,9,10]. Consequently, stem cells which encode for a treatment gene, e.g., CD, may form a less specific type of gene-directed EPT for tumors [5,12,13,14].

Previous study has demonstrated the migration of human neural stem cells encoding CD, which underwent systemic transplantation, towards a malignant lesion. In synergy with prodrug 5-FC, these cells induced a notable reduction in the volume of an implanted tumor [5]. Despite the fact that these human neural stem cells exhibited a marked capacity to drift towards the malignancy, this technique is limited by possible ethical constraints, the potential for reproducibility, and the low likelihood of being able to introduce these stem cells into the clinical domain.

A further strategy was therefore developed in order to resolve the above limitations regarding the traditional EPT combination of CD and 5-FU. This involved the use of an EPT system which comprised CD, 5-FC and adipose-derived stem cells (ADSC). The latter exploited the tumor tropism property of the stem cells to optimize therapeutic enzyme expression and distribution within the environment of the cancer, thereby inducing efficacious volume loss in tumor bulk whilst achieving a lower level of toxicity.

It is relatively straightforward to transduce ADSC using viral vectors, which offers the potential to attain enduring stem cell therapeutic gene expression. This, in turn, can facilitate these genes to be selectively targeted towards particular tumors in order to eradicate cancer cells [6]. In this study, immortalized human hTERT-ADSC were utilized to create ADSC with CD overexpression. This method of manipulating ADSC to express a suicide-gene EPT system is extremely safe for the delivery of gene therapy as, firstly, there is a bystander effect of eliminating cancer cells, and secondly, in vivo, the ADSC per se are also eradicated. The aim of the current research was to study the effects of bacterial cytosine deaminase-overexpressing human TERT-immortalized human adipose stem cells (hTERT-ADSC.CD) on the suppression of the growth of tumors, whilst exhibiting lower toxicity levels, in CRPC-bearing mice.

## 2. Results

### 2.1. Generation of CD from hTERT-ADSC

The immortalized human adipose mesenchymal stem cell line containing hTERT, ASC52TELO hTERT-ADSC (ATCC^®^ SCRC-4000TM) exhibits cell markers specific to mesenchymal stem cells, i.e., CD29, CD90 and CD105. CD35 and CD45 expression, typical of hematopoietic stem cells, is absent. In this study, a telomerase activity marker (Product sheet from ATCC^®^ SCRC-4000TM, ATCC, Manassas, VA, USA) was used as an assay for telomerase (Telomerase Repeat Amplification Protocol (TRAP)).

### 2.2. CD Protein Presence in hTERT-ADSC.CD

**Figure 1:**
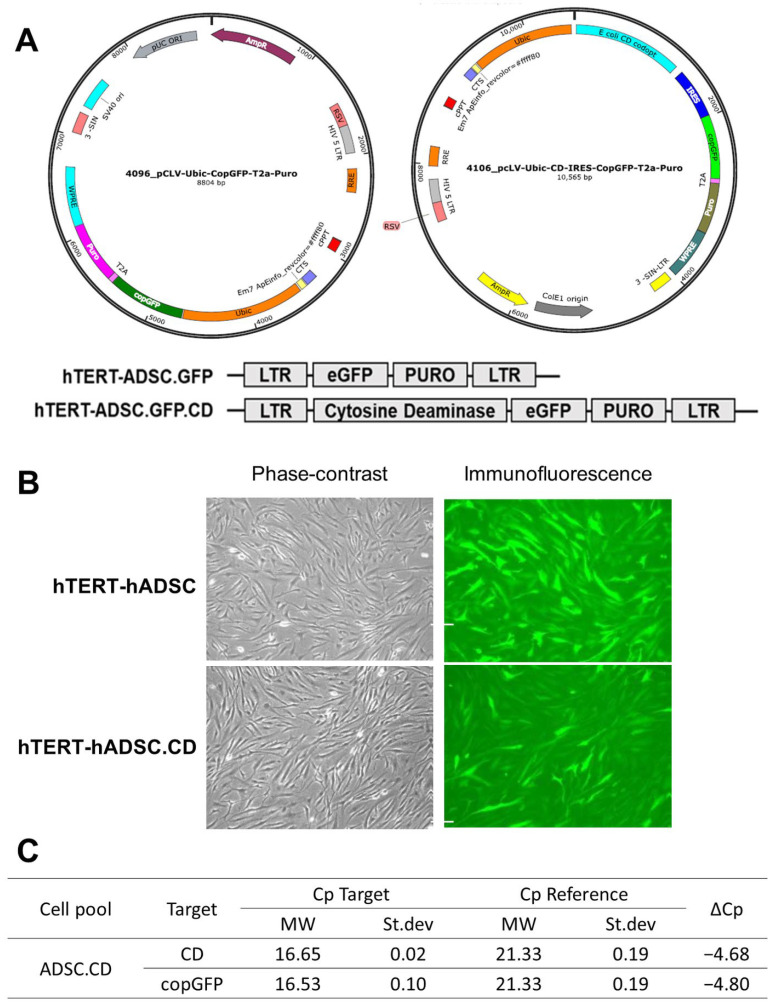
CD overexpressing hTERT-immortalized human adipose stem cell line (hTERT-hADSC.CD). (**A**): hTERT-hADSC and hTERT-hADSC.CD were generated via lentiviral transduction of *GFP*, *CD* gene using CLV-Ubic vector. (**B**): Phase contrast and immunofluorescence microscopy of hTERT-hADSC.GFP and hTERT-hADSC.GFP.CD cells (×400). (**C**): Real time PCR. ΔCp of hTERT-hADSC.CD was −4.68. ADCS = hTERT-hADSC, CD = cytosine Deaminase, RT-PCR = Reverse transcription-polymerase chain reaction.

**Figure 2:**
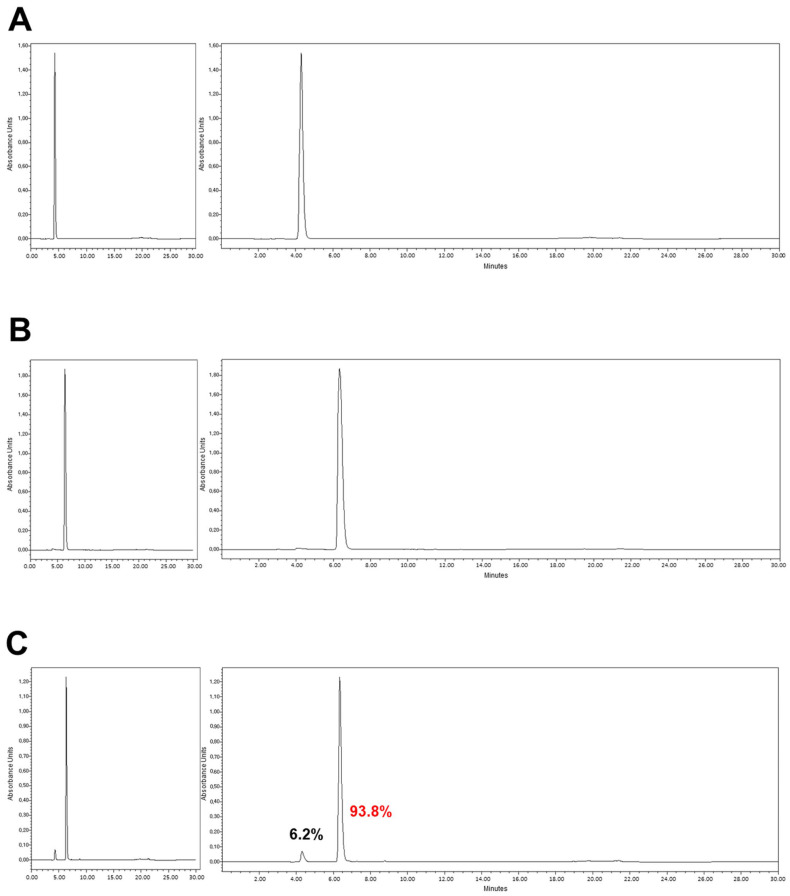
High-performance liquid chromatography (HPLC). HPLC analysis showed that 5-FC (**A**) and 5-FU (**B**) eluted at 4.3, 6.3 min, respectively. The percentages of 5-FC and 5-FU were 6.2 and 93.8 in hTERT-ADSC.CD (**C**). Flow = 1.0, Water(A)% = 100, MeCN(B)% = 0.

In order to create hTERT-ADSC.CD cells, the *CD* gene was introduced using lentiviral transduction with the lentiviral vector (CLV-Ubic) puromycin/CD vector (Figure 1). RT-PCR was used to verify hTERT-ADSC.CD CD transcript expression; ΔCp = −4.38. hTERT-ADSC.CD cells had a spindle appearance when viewed using phase contrast microscopy. Immunofluorescence microscopy revealed the presence of green fluorescent protein (GFP) positive cells which encompassed the hTERT-ADSC.CD *CD* gene (Figure 1). The high-performance liquid chromatography (HPLC) elution time for 5-FC was 4.3 min, and for 5-FU, 6.3 min; their respective proportions were 6.2% and 93.8%, indicating a 5-FC to 5-FU transformation yield of 93.8% when catalyzed by CD liberated from hTERT-ADSC and hTERT-ADSC.CD. HPLC was used to verify the existence of the CD Protein (Figure 2).

### 2.3. hTERT-ADSC.CD Phenotype and Its Ability

**Figure 3:**
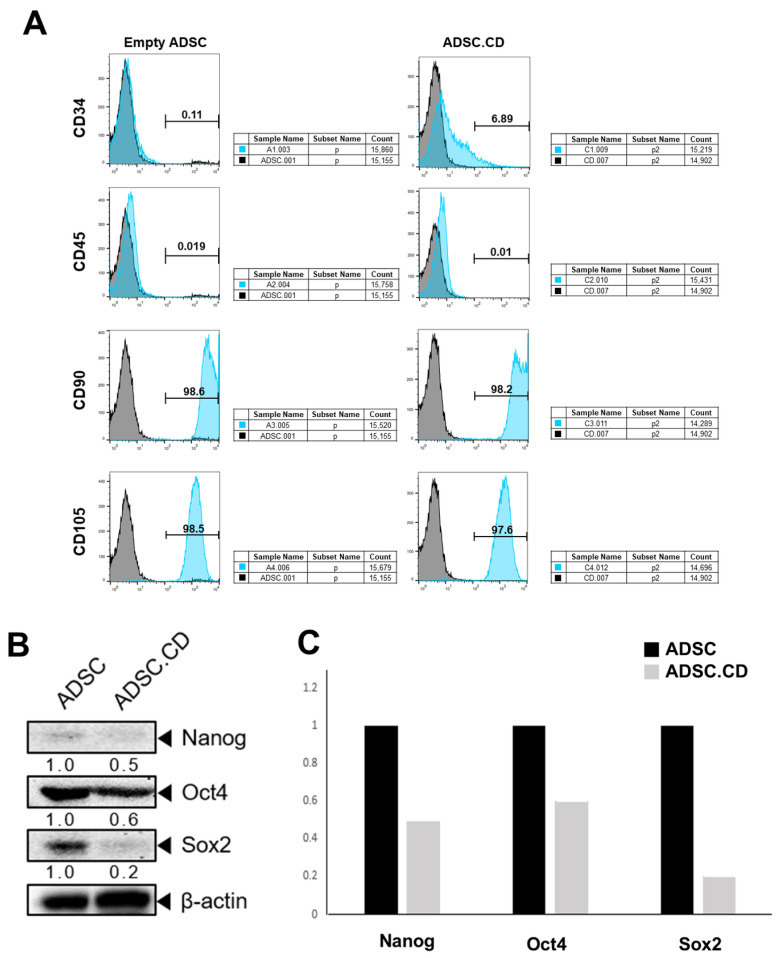
Cell markers for hTERT-hADSC.GFP and hTERT-hADSC.GFP.CD cells. (**A**): They express cell type-specific markers for mesenchymal stem cell markers CD29, CD90 and CD105, but not for hematopoietic stem cells CD34 or CD45. (**B**): Stem cell markers including Oct4, Nanog, Sox2 were expressed in hTERT-ADSC, hTERT-ADSC.CD cell line. (**C**): Quantitative expression of stem cell markers of in hTERT-ADSC, hTERT-ADSC.CD cell line. Maintenance of human MSCs was shown. hTERT-hADSC.CD = CD overexpressing hTERT immortalized human adipose stem cells. ADCS = hTERT-hADSC, ADSC.CD = CD overexpressing hTERT immortalized human adipose stem cells.

The expression of stem cell markers, such as Oct4, Nanog and Sox2, was identified in cells from the hTERT-ADSC and hTERT-ADSC.CD lineage. Human mesenchymal stem cell preservation was demonstrated (Figure 3).

**Figure 4:**
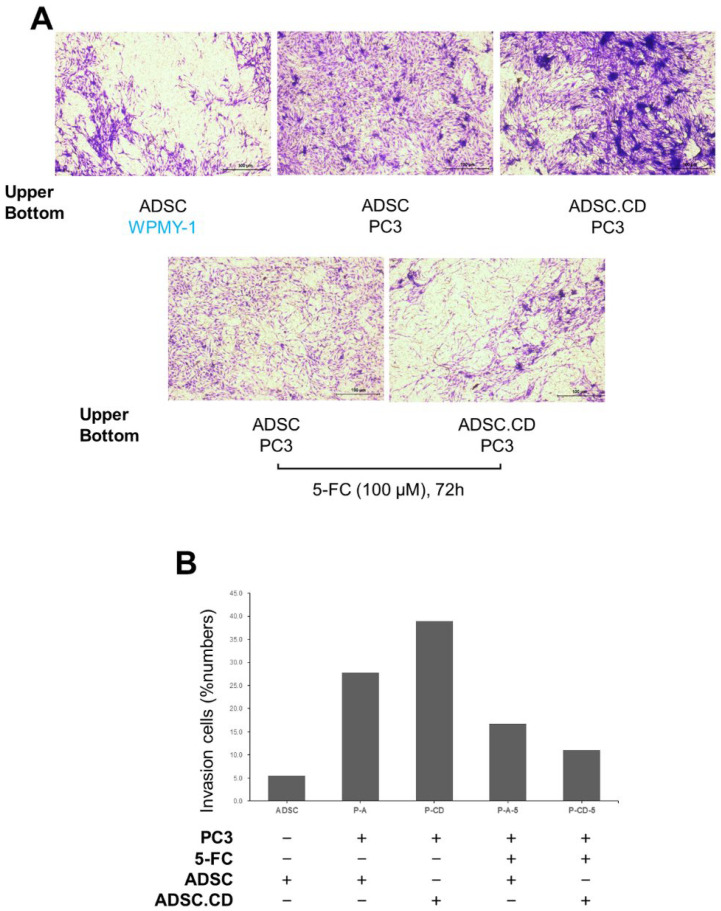
hTERT-ADSC.CD line targets prostate cancer. (**A**): Invasion study demonstrated the migration of hTERT-ADSC or hTERT-ADSC.CD to prostate cancer cells. (**B**): Quantitative expression of invasion study. ADCS = hTERT-hADSC, ADSC.CD = CD overexpressing hTERT immortalized human adipose stem cells.

In vitro studies demonstrated that hTERT-ADSC and hTERT-ADSC.CD were significantly induced to migrate in the direction of PC3 cells (*p* < 0.05) (Figure 4). A small number of hTERT-ADSC demonstrated drift toward WPMY-1 cells. The transgene expression of *CD* did not appear to influence the ability of the cells to migrate. 5-FC treatment diminished hTERT-ADSC.CD cell migration in the direction of the PC3 cells, although this tropism was more pronounced than that seen in the direction of WPMY-1 cells (*p* < 0.05) (Figure 4).

**Figure 5:**
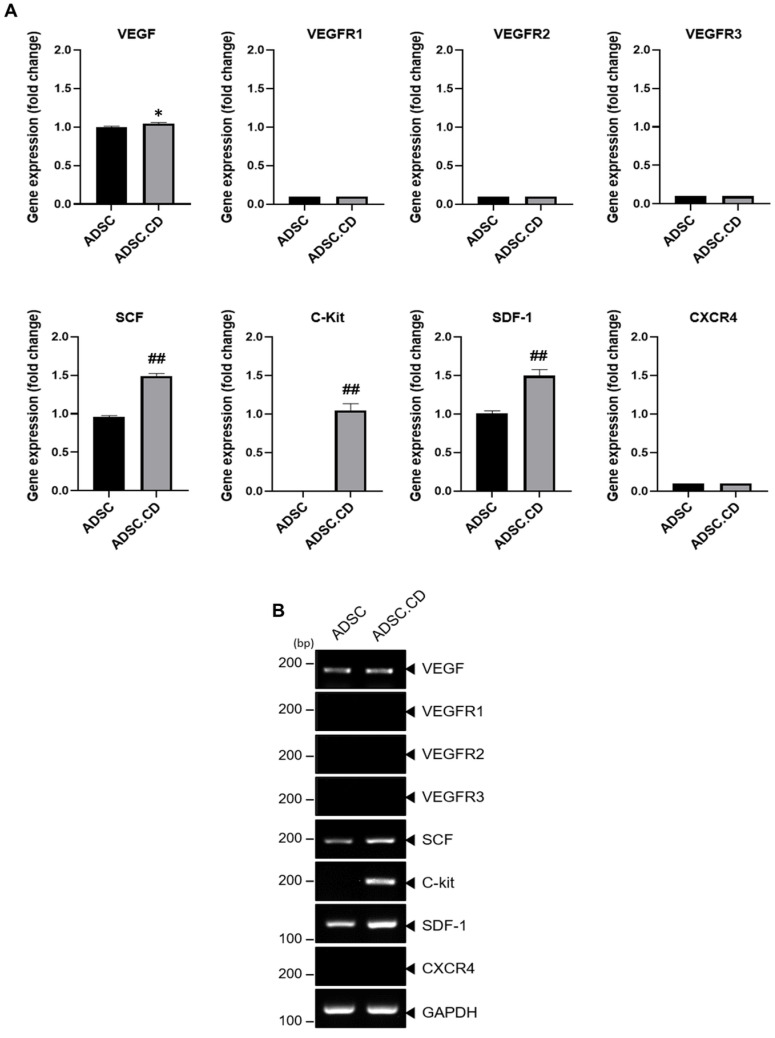
Graphical representation of migrant hTERT-ADSC.CD line. (**A**): Quantitative expression of chemoattractant factors. Using RT-PCR, the presence of ligands SCF, SDF-1 and VEGF and their corresponding receptors c-kit, in ADSC.CD cells, was confirmed. Using real-time PCR, the level of VEGF, SCF, SDF-1 c-kit increased. (**B**): RT-PCR of chemoattractant factors. ADCS = hTERT-hADSC, SCF = stem cell factor, SDF = stromal cell-derived factor, VEGF = vascular endothelial growth factor, VEGFR = vascular endothelial growth factor receptor. * *p* < 0.05. ^##^ *p* < 0.001. Data = mean ± standard error.

It was verified that the ADSC.CD contained ligands SCF, SDF-1 and VEGF and their relevant receptors, e.g., c-kit, by RT-PCR (Figure 5). The levels of these compounds were determined to be higher in the ADSC.CD than in the ADSC (*p* < 0.05) (Figure 5).

### 2.4. In Vitro Study

**Figure 6:**
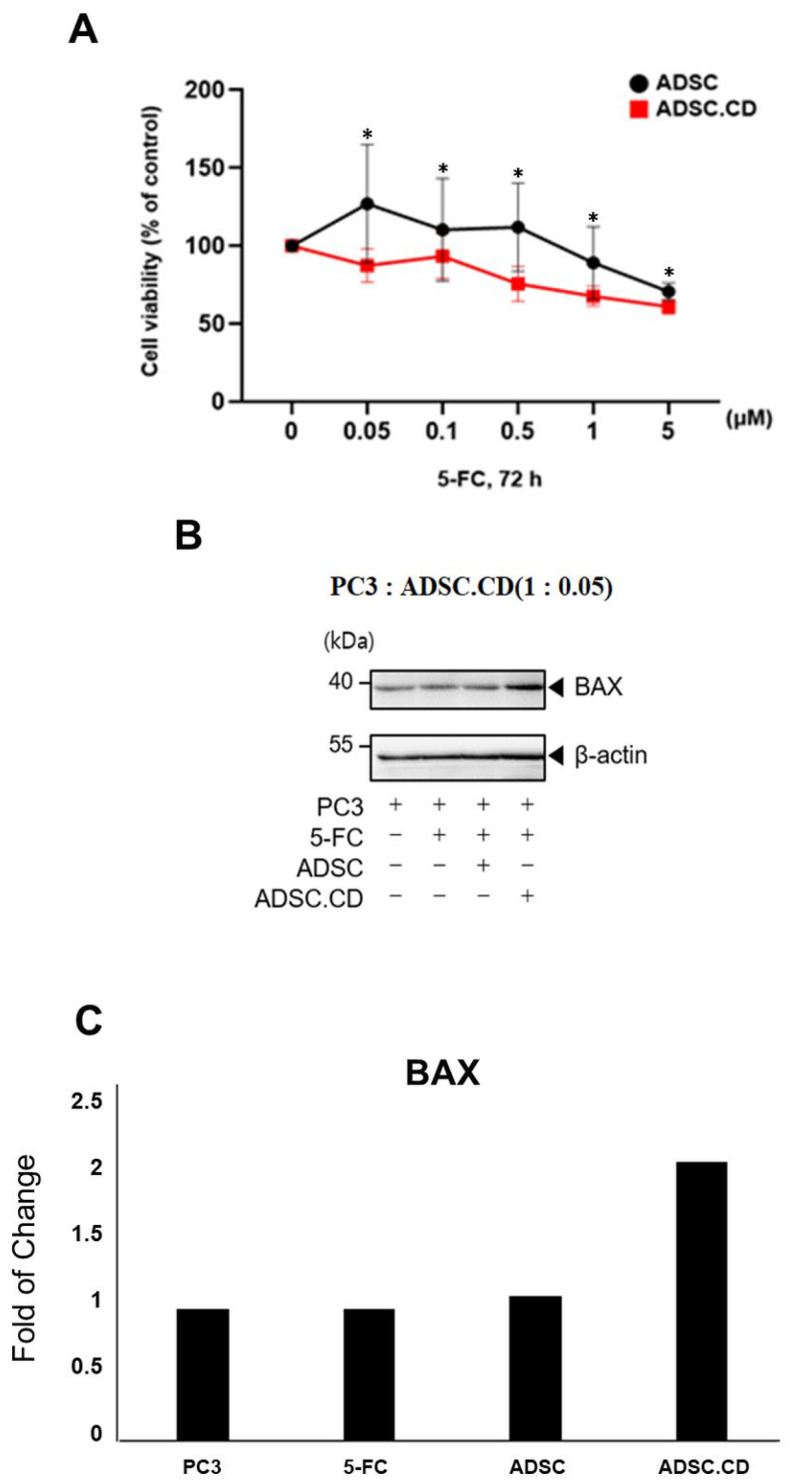
In vitro effect of hTERT-ADSC.CD under the treatment of 5-FC. (**A**): The suicide effect of hTERT-ADSC.CD was compared with that of hTERT-ADSC. At more than 0.05 μM 5-FC, the cell viability of ADSC.CD was lower than that of ADSC. The Mann–Whitney U test was used for statistical analysis. (**B**): The apoptotic effects of hTERT-ADSC.CD was compared with those of hTERT-ADSC or 5-FC monotherapy. Apoptotic PC3 cells increased in the presence of ADSC.CD under 5-FC compared with hTERT-ADSC under 5-FC or 5-FC monotherapy. (**C**) Quantitative analysis of the apoptotic effect. ADCS = hTERT-hADSC, ADSC.CE = CE overexpressing hTERT immortalized human adipose stem cells. * *p* < 0.05. Data = mean ± standard error.

The cellular viability of hTERT-ADSC.CD was lower than that of hTERT-ADSC between 0.05 and 5 µM/L 5-FC concentrations (*p* < 0.05) (Figure 6).

In the PC3 cells which demonstrated apoptosis and underwent treatment with hTERT-ADSC.CD and 5-FC, as opposed to 5-FC, hTERT-ADSC or PC3 as sole agents, elevated BAX levels and diminished BCL2 titers were evident in western blot analysis (Figure 6).

### 2.5. In Vivo Tumor Growth Inhibition of hTERT-ADSC.CD

**Figure 7:**
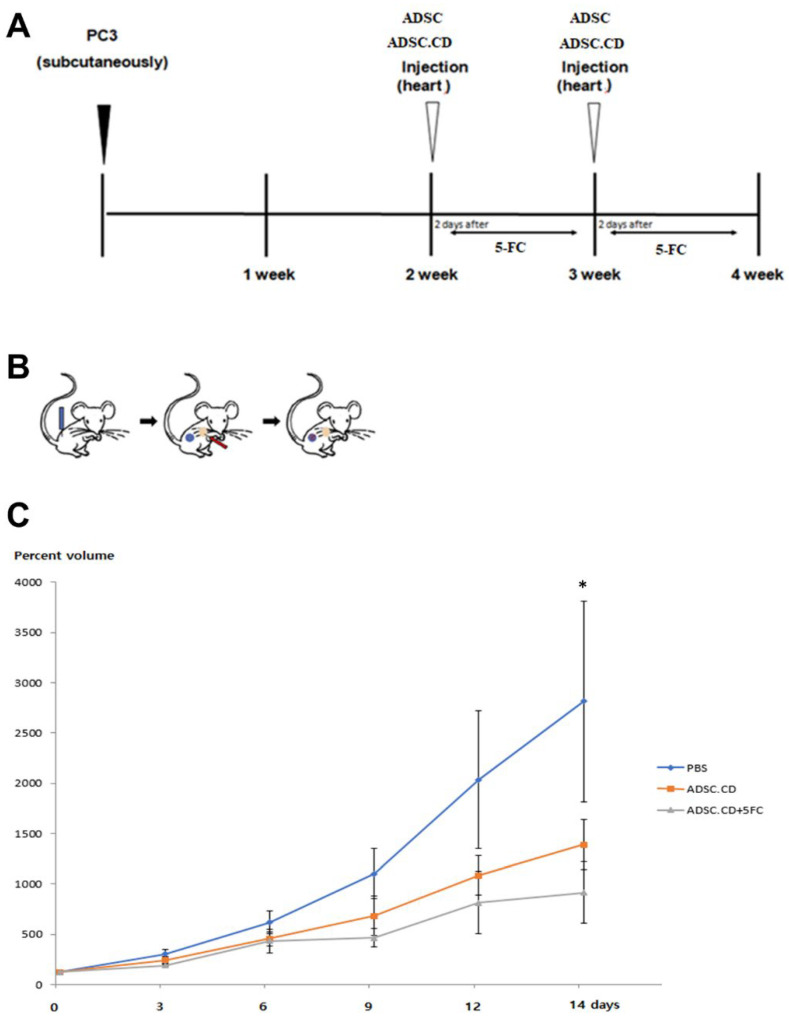
Treatment with hTERT-ADSC.CD cells and 5-FC has a significant therapeutic effect in vivo. (**A**): Schematic summary of the treatment. (**B**): Illustration of the induction of prostate cancer using PC3, systemic injection of hTERT-ADSC.CD cells and migration of the gene-modified stem cells toward the prostate cancer. Blue: PC3; red: hTERT-ADSC.CD cells. (**C**): The inhibitory effects of 5-FC showed better effects with ADSC.CD + 5-FC than those with ADSC.CD or 5-FC alone. hTERT-ADSC.CD = CD over-expressing hTERT immortalized human adipose stem cells, PBS = phosphate-buffered saline. * *p* < 0.05. Data = mean ± standard error.

The PC volumes (percent) seen after two weeks of systemic treatment in the different groups, compared with baseline, were as follows: hTERT-ADSC.CD and 5-FC, 888.4 ± 305.7; phosphate-buffered saline (PBS) only, 2786.7 ± 994.2; ADSC.CD only, 1365.3 ± 248.7. The group of hTERT-ADSC.CD in combination with 5-FC demonstrated more significant inhibitory effects on tumor growth than the PBS group (*p* < 0.05) (Figure 7). A decreased volume of PC was clearly evident in the animals treated with the combination of hTERT-ADSC.CD and 5-FC.

## 3. Discussion

In order to attain a more efficacious clinical endpoint following therapy, which was associated with a more favorable toxicity profile, a treatment approach comprising CD, 5-FC and ADSC (hTERT-ADSC.CD) was investigated in this study. The results indicate that this combination of agents has potential for treating CRPC (Figure 1). Using CD, together with 5-FC, is one of the more frequently utilized oncotherapeutic alternatives, particularly for the treatment of solid cancers. CD–5-FC is also often selected owing to its strong inhibitory activity with respect to cancer cells [3,4].

Earlier pre-clinical work from this laboratory has demonstrated that the systemic transplantation of human neural stem cells which included *CD* encoding (HB.1.F3.CD) evidenced migration along a vector toward malignant cells. When combined with 5-FC, a prodrug, this led to a marked reduction in the volume of the tumor [5].

Additional pre-clinical studies have also suppressed tumor volume enhancement in the absence of a virulent effect. An EPT system comprising HB1.F3.CD or HB1.F3.CD.IFN-β cells, together with 5-FC, was utilized with respect to human lymph node-derived metastatic colorectal adenocarcinoma cells (SW-620), ex vivo [7]. A further combination EPT system was investigated by Yi et al. [15], which comprised CD, human neural stem cells, 5FC and IFN-β (HB1.F3.CD and HB1.F3.CD.IFN-β). In LNCaP PC cells, an effective migration toward the tumor was observed, together with significant tumor growth suppression.

The current study involved the manufacture of hTERT-ADSC.CD using a lentiviral transduction of the *CD* gene, achieved with a CLV-Ubic puromycin/CD vector (Figure 1). hTERT-ADSC and hTERT-ADSC.CD had a spindle appearance under phase contrast microscopy. This finding demonstrated human mesenchymal cell preservation. Since transfection of the complete coding sequence for the *E. coli CD* gene was carried out, it was not possible to attain the corresponding anti-CD antibody. Although western blot analysis was unable to be used to verify CD protein expression from hTERT-ADSC.CD as a consequence, this was demonstrated using RT-PCR. A value of ΔCp, −4.38 was obtained for hTERT-ADSC.CD, and the degree of CD expression change was enhanced. Immunofluorescence microscopy also indicated the *CD* gene in the *GFP* positive cell, confirming *CD* transcript expression (Figure 1). *CD* and *GFP* gene were transfected together, GFP positive cells have *CD* gene also.

The percentages of 5-FC and 5-FU attained following HPLC analysis were 6.2% and 93.8%, respectively. This result indicated that the CD enzyme, liberated from the hTERT-ADSC.CD had catalyzed the transformation of 5-FC to 5-FU by this proportion. HPLC analysis also indicated that CD protein existed in the hTERT-ADSC.CD (Figure 2).

CD29, CD90 and CD105 mesenchymal stem cell markers were expressed by the hTERT-ADSC.CD cells, but there was no hematopoietic stem cell marker expression, e.g., of CD34 or CD45. The stem cell characteristics, therefore, remained unchanged following the introduction of CD into hTERT-ADSC. Cells of hTERT-ADSC or hTERT-ADSC.CD lineage expressed the Oct4, Nanog and Sox2 stem cell markers, indicating preservation of human mesenchymal stem cells (Figure 3).

The bacterial *CD* gene, obtained from *E. coli*, is a popular suicidal gene, which has been demonstrated to suppress the growth of cancer cells. The *CD* gene encodes an enzyme which promotes the formation of uracil from cytosine. A hydrophilic pharmaceutical agent, with anti-fungal properties, 5-FC has a favorable toxicity profile in humans. The prodrug 5-FC is transformed into 5-FU, which has anti-cancer effects, by CD. This catalytic activity of CD has led to the investigation of this enzyme as a candidate for use in the gene therapy of solid malignancies.

The presence of the ligand VEGF, which had tropic chemo-attractant properties, and the receptor VEGF-1 (Flt1), were also confirmed [9].

The carrier for the EPT system in the present work was ADSC, which can be accessed without difficulty and in the absence of any ethical consternation regarding the origin of the stem cells. Additionally, ADSC can be proliferated ex vivo to the necessary volume for genetic alterations [10]. It is simple to acquire ADSC from the patient receiving the treatment, and as undifferentiated cells, an immune response is not activated. Additionally, this method circumvents the likelihood of immune rejection. The process of human ADSC gene manipulation can be carried out efficaciously, and the cell population swiftly increased to that necessary for clinical use.

ASC52TELO hTERT-ADSC (ATCC^®^ SCRC-4000TM) are immortalized human cells from the adipose mesenchymal stem cell lineage, and include hTERT (Figure 1). The acquisition of ADSC requires an anesthetic, and they can be challenging to store, an issue that can be resolved by their immortalization. When ADSC are adapted by the inclusion of hTERT, the latter is not carcinogenic, and so the final product, the immortalized hTERT-ADSC, can act as a carrier for therapeutic gene transduction [16]. Since migration in the direction of a tumor is an intrinsic property of stem cells, a potentially favorable cell-based delivery approach is to incorporate relevant genes within them [11,12,13]. The generation of malignant lesion stroma is a driving force behind stem cell tropism and incorporation within the tumor lesion; there is marked stem cell replication as the malignancy develops [13]. The in vitro experiments in this study showed that the PC3 cells (*p* < 0.05) acted as triggers for the migration of the hTERT-ADSC.CD cells in their direction (Figure 4).

Migration of hTERT-ADSC.CD to the location of the neoplasm can diminish the volume of the tumor. Consequently, in this study it was anticipated that hTERT-ADSC.CD would demonstrate migration towards the PC cells and exhibit anti-tumor activity as a result of the incorporated suicide genes.

In the current work, the ADSC.CD were shown to incorporate ligands and their receptor counterparts, i.e., SCF, SDF-1 and VEGF, and c-kit, respectively. Their concentrations in the ADSC.CD population were greater (*p* < 0.05) than measured in the ADSC (Figure 5).

The extremely toxic metabolic by-products arising from the CD-catalyzed 5-FC to 5-FU reaction led to the reduction of the ADSC, which consequently avoided cancer-inducing effects arising from the ADSC. More enduring 5-FU synthesis and more concentrated regional titers occurring as a result of the bystander effect enhanced treatment efficacy (*p* < 0.05) in this study (Figure 6).

Transformation of the prodrug was achieved through alterations in the enzyme in order to maximize the cytotoxic effect. In the current research, *CD* gene expression lacked toxicity, and a similar growth ratio to the parental cell population was seen in the *CD*-transduced cells. Nevertheless, in vitro, the latter (hTERT-ADSC.CD) were reducted, and in contrast to their parental hTERT-ADSC, appeared more susceptible to the effects of 5-FC (Figure 6). Following 5-FC therapy, the suicide effect caused by hTERT-ADSC.CD was confirmed. PC3 cells undergoing apoptosis demonstrated elevated BAX in western blot analysis; diminished BCL2 levels were seen in hTERT-ADSC.CD in comparison to those treated with 5-FC, hTERT-ADSC or PC3 as sole agents (Figure 6). Apoptosis was promoted in PC3 cells administered both hTERT-ADSC.CD and 5FU.

Injecting into the left ventricle of heart means that major stem cell distribution to the capillary bed in the lungs was circumvented [17,18]. The systemic delivery of hTERT-ADSC.CD, together with 5-FC, led to a greater reduction in PC volume compared with that seen in control mice or those given 5-FC alone (*p* < 0.05) (Figure 7).

An ex vivo comparative pre-clinical study of mesenchymal stem cells which exhibited four different suicide genes, i.e., two variants of thymidine kinase, CD and NTR, was carried out by Nouri et al. [19]. The EPT system combination with the greatest therapeutic impact on SKOV3 ovarian tumors was the one comprising CD and 5-FC. However, it is not easy to determine the degree to which a particular EPT component or combination thereof is responsible for the overall treatment effect, owing to the broad spectrum of malignancies, carriers used, and the prodrug and pathological context. Ongoing pre-clinical research is therefore essential in order to demonstrate the advantages and disadvantages of each therapeutic option investigated in order to ascertain specific prospective clinical endpoints [3,4,5,8,9,20].

The current work demonstrated that *CD*-transduced human ADSC, in combination with 5-FU, exhibited cytotoxicity towards implanted PC cells. The findings substantiate the utilization of amplified CD expression in human ADSC as a therapeutic option for late-stage PC in clinical trials.

## 4. Materials and Methods

### 4.1. Cell Culture

Following procurement, ASC52TELO hTERT-ADSC (ATCC^®^ SCRC-4000TM, ATCC, Manassas, VA, USA) were incubated in Dulbecco’s modified Eagle medium (DMEM, GibcoBRL, Grand Island, NY, USA), together with 2 mM L-glutamine, 100 U/mL penicillin, 100 µg/mL streptomycin and 10% heat inactivated fetal bovine serum (FBS, GibcoBRL, Grand Island, NY, USA). Cells from the PC line PC3 (Korean cell line bank, Seoul, Republic of Korea) were cultured in the same medium with identical supplements. Conditions for culture included: air, 95%; carbon dioxide, 5%; temperature, 37 °C; and humidification. Cell passaging was achieved using trypsin.

### 4.2. Generation of hTERT-ADSC.CD Lines

The lentiviral vector CLV-Ubic/CD, which included the entire coding sequence of the bacterial *CD* gene from *Escherichia coli* (Gene ID 3096544), was used to create the recombinant lentivirus (Figure 1). Calcium phosphate co-precipitation was used to achieve transfection. The medium was substituted the next day, and after a further 16–20 h, the supernatant, which formed the recombinant lentivirus stock solution, was decanted.

Following lentovirus infection, cells were incubated in 8 µg/mL polybrene (Sigma, St. Louis, MO, USA) for between 4 and 6 h at a temperature of 37 °C. Following medium substitution with a de novo virus-free medium, cells were cultured at 37 °C for 48 h. An amount of 3 µg/mL puromycin (Sigma, St. Louis, MO, USA) was added to facilitate the identification of infected cells. Those exhibiting puromycin resistance underwent expansion for later experiments. Establishment of hTERT-ADSC.CD, carrying the *E. coli CD* gene (Gene ID 3096544), was achieved.

### 4.3. High-Performance Liquid Chromatography: Identification and Quantitation 

The solution strengths of 5-FC and 5-FU were quantified. Prior to HPLC, molecules were transformed into a lactone form by acidification. Acidified methanol, comprising 5 µL 1 N HCI/mL methanol, was used to dilute the samples. These underwent centrifugation (14,000× *g*, 2 min). The resulting supernatant was passed at a flow rate of 1 mL/min through a 4-p,m Nova-Pak C,8 column, of dimensions 300 × 3.9 mm, for which 75 mM ammonium acetate, 25% acetonitrile (pH 4.0) had been used for equilibration. Elution of 5-FC occurred under these parameters after 4.3 min, and 5-FU, after 6.3 min. A Jasco 82 1-FP fluorescence detector was utilized for product identification, using excitation and emission wavelengths of 375 nm and 550 nm, respectively. System Gold software (32 Karat Software, version 3.0) was employed for data analysis. Detection limits were 20 pg/pA for 5-FC and 2 pg/pA for 5-FU.

### 4.4. Analysis of hTERT-ADSC.CD

#### 4.4.1. RT and Real-Time PCR

TRIzol reagent (GIBCO-BRL, Life Technologies, Grand Island, NY, USA) was utilized for total RNA extraction from specified cell clones. A random primer was used in combination with the total RNA for reverse transcription. The primers used for real-time polymerase chain reaction (RT-PCR) analysis were: CD, VEGF, VEGFR1, VEGFR2, VEGFR3, SCF, c-kit, SDF-1 and CXCR4 (Table 1). Equal protein loading was demonstrated by GAPDH controls. The following successive stages comprised the amplification process: 3 min, denaturation; 1 min, 35 cycles at 95 °C; 1 min, 35 cycles at 63 °C; and 1 min, 35 cycles at 72 °C. Electrophoresis using 1.2% agarose 1X TAE gels, together with ethidium bromide stain, was used to analyze the yield from the amplification. Table 1 presents the chemoattractant ligands and receptors, as indicated by the sense and anti-sense primers, respectively, as well as the predictions for the RT-PCR reaction product sizes.

#### 4.4.2. Western Blot Analysis

Extraction of all the proteins was accomplished with the use of RIPA lysis buffer (ThermoFisher Scientific, Waltham, MA, USA). These were then isolated by electrophoresis on a sodium dodecyl sulfate-polyacrylamide gel, and then relocated to polyvinylidene fluoride membranes (Millipore, Billerica, MA, USA) in order to perform western blot analysis. The membranes were blocked with 5% skimmed milk, and then incubated with primary antibodies specifically against Nanog, Oct4, Sox2, BAX, cleaved caspase-3, BCL2, β-actin (1:3000, Santa Cruz Biotechnology, Dallas, TX, USA), and subsequently, secondary antibodies conjugated with peroxidase. An enhanced chemiluminescence reagent (Amersham Biosciences, Little Chalfont, Buckinghamshire, UK) was used to visualize the bands for analysis, and a camera was utilized for cell image acquisition. A system for image analysis was employed in order to quantify the findings (National Institutes of Health [NIH] Image J 1.34, http://rsbweb.nih.gov/ij/, accessed on 1 December 2023).

#### 4.4.3. Flow Cytometry Analysis of hTERT-ADSC.CD

Flow cytometry was conducted using Cyflow Cube 8 FACS hardware version 1.6 (SysmexPartec, Görlitz, Germany); FSC Express software, version 7.08.0018 (De Novo Software, Los Angeles, CA, USA) was used for data analysis. Antibodies against CD29, CD90 and C105 (1:50, Origene Technologies, Rockville, MD, USA), CD 34 (1:50, Invitrogen, Carlsbad, CA, USA) and CD 45 (1:50, LDBio, Seattle, WA, USA) were utilized for hTERT-ADSC and hTERT-ADSC.CD flow cytometry analysis.

The immunostained cells underwent analysis using two-color flow cytometry (BD FACS Canto II; BD, Franklin Lakes, NJ, USA). A camera was used for image acquisition. The stained cell percentage was computed by data comparison with the equivalent negative control sample. Processing the images using an image analyzer system provided quantitative data (National Institutes of Health [NIH] Image J 1.34, http://rsbweb.nih.gov/ij/, accessed on 1 December 2023).

### 4.5. Cell Invasion Assays of hTERT-ADSC.CD toward Prostate Cancer

The migration of hTERT-ADSC.CD towards PC cells was evaluated in Matrigel-coated transwell cell culture chambers with a pore size of 8 μm (Merck Millipore, Billerica, MA, USA). At 24 h prior to commencing the experiment, 1.5 × 10^4^ cells of human prostatic myofibrosblast lineage, WPMY-1, or alternatively, medium, were added to the 24-well plates in the lower well. 105 RT-ADSC and hTERT-ADSC.CD cells, suspended in a serum-free medium were added to the ECMatrix inserts (pore size, 8 Am). PBS was used to rinse the cells in the lower wells. Serum-free medium was then added, and then the wells were placed in an incubator for 48 h for the migration assay. ECMatrix, together with any non-invading cells, was removed from the insert interior. Following 20 min exposure to the stain, photographs were captured microscopically. The abundances of migrated cells within 5 regions of interest were counted, and the mean computed. Images of cells were captured with a camera. An image analyzer system was used to obtain quantitative data (National Institutes of Health [NIH] Image J 1.34, http://rsbweb.nih.gov/ij/, accessed on 1 December 2023).

### 4.6. Confirmation of Chemoattractant Ligands and Receptors

Ligand stem cell factor (SCF), stromal cell-derived factor 1 (SDF-1) and vascular endothelial growth factor (VEGF) transcription in ADSC and ADSC.CD, together with the transcription of their associated receptors, i.e., c-kit, chemokine receptor 4 (CXCR4), VEGF receptor (VEGFR)-1, VEGFR2 and VEGFR3 in the ADSC.CD, were evaluated using RT-PCR. Total RNA, in association with a random primer, was used for reverse transcription. Equal protein loading was affirmed with the use of GAPDH controls. The following successive stages comprised the amplification process: 3 min, denaturation; 1 min, 35 cycles at 95 °C; 1 min, 35 cycles at 63 °C; and 1 min, 35 cycles at 72 °C. Electrophoresis using 1.2% agarose 1X TAE gels, together with ethidium bromide stain, was carried out to analyze the amplification output. Table 1 presents the chemoattractant ligands and receptors, as indicated by the sense and anti-sense primers, respectively, as well as the predictions for the RT-PCR reaction product sizes. A camera was utilized to obtain cell images, which were then subjected to quantitative analysis using an image analyzer system (National Institutes of Health [NIH] Image J 1.34, http://rsbweb.nih.gov/ij/, accessed on 1 December 2023).

### 4.7. In Vitro Analysis of hTERT-ADSC.CD 

#### 4.7.1. Suicide Effect of hTERT-ADSC.CD/5-FC System

To evaluate hTERT-ADSC.CD sensitivity to 5-FC, 96-well plates (Falcon, Becton-Dickinson Co., Flanklin Lakes, NJ, USA) were utilized. Each experiment was repeated 4 times. A total of 5 × 10^3^ cells were added to each well; concentrations ranging from 0.05 to 5 μM 5-FC were added after 24 h. The well plates were placed in an incubator for 72 h at a temperature of 37 °C. Quantification of cell viability was achieved with the use of a modified MTT assay (Promega, Madison, WI, USA), which indicated transformation of the MTT tetrazolium salt to the formazan moiety, a reaction catalyzed by mitochondrial dehydrogenase. Then, 10 μL MTT solution was pipetted into the individual wells and the plate incubated at 37 °C for 4 h. Dimethyl sulfoxide (Sigma Aldrich, St. Louis, MO, USA) was added at the same temperature for 20 min for color extraction. A microplate reader (Infinite F50; Tecan, Männedorf, Switzerland) was used to measure the reaction’s absorbance at a wavelength of 570 nm, which enabled quantification of the formazan product. Measurements of cell viability were calculated as a proportion of the control viability and given as mean ± standard error (SE).

#### 4.7.2. Apoptotic Effect of hTERT.ADSC.CD/5-FC System

The cell fraction displaying apoptosis was established using western blot analysis. BAX, cleaved caspase-3 and BCL2 were used for hTERT-ADSC.CD analysis. Following image acquisition with a camera, quantification was performed using an image analyzer system (National Institutes of Health [NIH] Image J 1.34, http://rsbweb.nih.gov/ij/, accessed on 1 December 2023).

### 4.8. In Vivo Tumor Growth Inhibition on Systemic Administration of hTERT-ADSC.CD

The National Institute of Health Guide for the Care and Use of Laboratory Animals (2001) was followed during all experiments. Ethical approval had been obtained from the institution’s Institutional Animal Care and Use Committee (Soonchunhyang University Hospital, Seoul, IRB no:2019-4). The adult male nude mice, with bodyweight range 20–25 g, were purchased from OrientBio (Seognam, Republic of Korea). The mice were assigned to one of 3 cohorts: (i) control mice, who received no treatment (*n* = 5); (ii) mice treated with ADSC.CD only (*n* = 5); and (iii) mice treated with ADSC.CD and 5-FC (*n* = 5). The environment in which the mice were kept was temperature-regulated and had a 12 h light: 12 h dark cycle. They were fed normal food and had unrestricted water access. Isoflurane (BKPharm, Goyang, Republic of Korea) was used for anesthesia, and a 30-gauge Hamilton syringe was used to deliver 1.0 × 10^6^ PC3 cells suspended in 100 μL PBS into the flank subcutaneous tissue. At 7 and 14 days after the intracardiac delivery of PBS, hTERT-ADSC or hTERT-ADSC.CD, a treatment solution comprising 500 μg/kg/day 5-FC in 100 μL PBS was given intraperitoneally, over 2 periods of 5 consecutive days separated by a 2-day interval. A caliper was used to measure the tumor on day 0 and day 14 following injection. Calculation of the tumor volume was performed using the formula: volume = (length × width^2^)/2. The animals were killed on day 14 following injection, and the tumors resected. Data for the tumor volume were expressed as mean ± SE.

### 4.9. Statistical Analysis

The significance of any differences with respect to cell viability was evaluated using the Mann–Whitney U test, and the volume of the tumor was evaluated using two-way analysis of variance and the post-hoc Tukey test. All data are expressed as mean ± SE. Statistical significance was defined as a *p*-value < 0.05.

## 5. Conclusions

hTERT-ADSC, in which *CD* gene had been incorporated, demonstrated the capacity to migrate in the direction of PC, and, in vivo, markedly suppressed tumor growth when given together with the prodrug 5-FC. This de novo EPT system, which exploits the individual’s own ADSC, offers an innovative clinical approach for the treatment of CRPC.

## Figures and Tables

**Table 1:** PCR Primer Sequences.

Gene	Sequence	Size (bp)
*CD*	Sense: 5′-GCGCGAGTCACCGCCAGCCACACCACGGC-3′	559
	Antisense: 5′-GTTTGTAATCGATGGCTTCTGGCTGC-3′	
*SCF*	Sense: 5′-ACTTGGATTCTCACTTGCATTT-3′	505
	Antisense: 5′- CTTTCTCAGGACTTAATGTTGAAG-3′	
*c-kit*	Sense: 5′- GCCCACAATAGATTGGTATTT-3′	332
	Antisense: 5′-AGCATCTTTACAGCGACAGTC-3′	
*SDF-1*	Sense: 5′-ATGAACGCCAAGGTCGTGGTC-3′	200
	Antisense: 5′-GGCTGTTGTGCTTACTTGTTT-3′	
*CXCR4*	Sense: 5′-CTCTCCAAAGGAAAGCGAGGTGGACAT-3′	733
	Antisense: 5′-AGACTGTACACTGTAGGTGCTGAAATCA-3′	
*VEGF*	Sense: 5′-AAGCCATCCTGTGTGCCCCTGATG-3′	541
	Antisense: 5′-GCTCCTTCCTCCTGCCCGGCTCAC-3′	
*VEGFR1*	Sense: 5′-GCAAGGTGTGACTTTTGTTC-3′	512
	Antisense: 5′-AGGATTTCTTCCCCTGTGTA-3′	
*VEGFR2*	Sense: 5′-ACGCTGACATGTACGGTCTAT-3′	438
	Antisense: 5′-GCCAAGCTTGTACCATGTGCG-3′	
*VEGFR3*	Sense: 5′-AGCCATTCATCAACAAGCCT-3′	298
	Antisense: 5′-GGCAACAGCTGGATGTCATA-3′	
*GAPDH*	Sense: 5′-CATGACCACAGTCCATGCCATCACT-3 Antisense: 5′-TGAGGTCCACCACCCTGTTGCTGTA-3′	450

## Data Availability

The data used and/or analyzed in this study are available on request from the corresponding author (Y.S.S.).
